# Predicting the Poor Clinical and Radiographic Outcomes after the Anatomical Reduction and Internal Fixation of Posterior Wall Acetabular Fractures: A Retrospective Analysis

**DOI:** 10.3390/jcm11113244

**Published:** 2022-06-06

**Authors:** Sung-Yen Lin, Cheng-Jung Ho, Wen-Chih Liu, Jr-Kai Chen, Hung-Pin Tu, Tien-Ching Lee, Je-Ken Chang, Chung-Hwan Chen, Cheng-Chang Lu

**Affiliations:** 1 Department of Orthopedics, Kaohsiung Medical University Hospital, Kaohsiung Medical University, Kaohsiung 80708, Taiwan; tony8501031@gmail.com (S.-Y.L.); rick_free@mail2000.com.tw (C.-J.H.); andysirliu@gmail.com (W.-C.L.); jkchang@kmu.edu.tw (J.-K.C.); 2 Department of Orthopedics, School of Medicine, College of Medicine, Kaohsiung Medical University, Kaohsiung 80708, Taiwan; tn916943@gmail.com (T.-C.L.); hwan@kmu.edu.tw (C.-H.C.); 3 Orthopedic Research Center, Kaohsiung Medical University, Kaohsiung 80708, Taiwan; 4 Regeneration Medicine and Cell Therapy Research Center, Kaohsiung Medical University, Kaohsiung 80708, Taiwan; 5 Department of Orthopedics, Changhua Christian Hospital, Changhua 50006, Taiwan; u9101046@hotmail.com; 6 Department of Public Health and Environmental Medicine, School of Medicine, College of Medicine, Kaohsiung Medical University, Kaohsiung 80708, Taiwan; p915013@cc.kmu.edu.tw; 7 Department of Orthopedics, Kaohsiung Municipal Ta-Tung Hospital, Kaohsiung 80145, Taiwan; 8 Ph.D. Program in Biomedical Engineering, College of Medicine, Kaohsiung Medical University, Kaohsiung 80701, Taiwan; 9 Department of Healthcare Administration and Medical Informatics, Kaohsiung Medical University, Kaohsiung 80701, Taiwan; 10 Institute of Medical Science and Technology, National Sun Yat-sen University, Kaohsiung 80420, Taiwan; 11 Graduate Institute of Animal Vaccine Technology, College of Veterinary Medicine, National Pingtung University of Science and Technology, Pingtung 91201, Taiwan; 12 Department of Orthopedics, Kaohsiung Municipal Siaogang Hospital, Kaohsiung Medical University, Kaohsiung 81267, Taiwan

**Keywords:** acetabular dome, anatomical reduction and internal fixation, fracture comminution, osteoarthritis, osteonecrosis, posterior wall acetabular fracture

## Abstract

Anatomical reduction is the fundamental principle of hip function restoration after posterior acetabular wall fractures (PWFs). Some patients exhibit poor outcomes despite anatomical reduction, and the prognostic factors leading to poor outcomes remain elusive. This study aimed to investigate the clinical and radiographic outcomes in patients with PWFs who had undergone anatomical reduction and internal fixation and to identify the predictors that impair clinical and radiologic outcomes. The clinical records of 60 patients with elementary PWFs who had undergone anatomical reduction and internal fixation between January 2005 and July 2015 were reviewed retrospectively. The Harris hip score (HHS) and modified Merle d’Aubigné clinical hip scores (MMAS) were used to evaluate the clinical outcome. Preoperative and final follow-up radiographs were cross checked to identify poor radiographic outcomes that included the presence of advanced osteoarthritis and osteonecrosis, as well as the need for conversion to total hip arthroplasty. Acetabular dome comminution was assessed from computerized tomography, and the outcomes were further evaluated according to the involvement of fragment comminution. The fracture comminution and age were negatively correlated with functional outcomes (correlation coefficients were −0.41 and −0.39 in HHS and MMAS, respectively) and were significantly related to the severity of osteoarthritis and osteonecrosis as well as the need for total hip arthroplasty. Regarding the radiographic factors, significantly worse post-operative HHS and MMAS were found in the fracture comminution group. In the subanalysis of the status of fracture comminution, patients with fragment comminution involving the acetabular dome had significantly lower functional scores than those with other fracture patterns. In conclusion, age, fracture comminution, and dome comminution were the prognostic indicators of advanced osteoarthritis and poor functional scores after the anatomical reduction and internal fixation of PWFs. We emphasized the relevance of acetabular dome comminution as an important contributing factor to clinical and radiographic outcomes.

## 1. Introduction

Posterior wall acetabular fracture (PWF) is the most common acetabular fracture, accounting for approximately one-third of all acetabular injuries [1]. The integrity of the posterior wall is mandatory for maintaining the stability of the hip joint, and open reduction and internal fixation (ORIF) is usually recommended in the presence of joint instability or incongruity [2]. The anatomical reduction of the articular surface and its stable fixation for early mobilization are widely propagated as the gold standards for the treatment of PWFs. Moreover, fracture reduction accuracy has been considered to be associated with long-term functional and radiological outcomes [3,4,5]. Total hip arthroplasty (THA) is a reliable surgical procedure following joint arthritis after the failed treatment of acetabular fractures. However, the significant bone loss and soft tissue adhesion in patients after prior ORIF of acetabular fractures are associated with a higher complication rate in terms of subsequent periprosthetic joint infection and dislocation than in patients undergoing primary THA [6,7].

Unexpected incarcerated bone fragments and marginal cancellous impactions, which are usually encountered and affect surgical reduction during ORIF, are usually not found on routine plain radiographs. Preoperative computed tomography (CT) scans help to comprehensively overview the complexity of PWF, such as fragment comminution, marginal cancellous bone impaction, femoral head injury, and intra-articular fragments, and therefore helps in surgical planning. It is difficult to obtain anatomical reduction and maintain the stability of PWF, even with advanced radiological examinations, owing to the complexity of fracture patterns and patient conditions.

Several authors have proposed different surgical techniques for achieving stable fixation. Giannoudis et al. used a two-level reconstruction technique for comminuted PWFs and reported favorable clinical outcomes, with excellent results in 72% of cases [8]. Kim et al. used a similar technique and reported satisfactory results in 60.7% and 72.7% of clinical and radiological outcomes, respectively [9]. Lee et al. advocated the spring plate technique for adjuvant fixation in comminuted PWFs and reported excellent to good results in 92.3% of cases radiologically, and only 4 of 52 patients required conversion to THA [10]. Despite the advances in imaging and surgical techniques, there is an apparent disparity in clinical outcomes and reduction quality. To date, there has been no study discussing the postoperative outcomes of anatomical reduction in PWF patients. In this study, we aimed to investigate clinical and radiological outcomes in patients with PWFs after anatomical reduction and internal fixation and to identify the predictors that impair clinical and radiological outcomes.

## 2. Materials and Methods

### 2.1. Participants

This study was approved by the institutional review board of our hospital. After approval, the medical records of patients with acetabular fractures admitted to the hospital over 10 years (January 2005 to July 2015) were retrospectively reviewed. The inclusion criteria were (1) age > 18 years; (2) acetabular fracture without any other combined fractures; (3) displaced PWFs (>2 mm) confirmed by radiography and CT, requiring surgical fixation as evaluated by an experienced hip surgeon, and classified according to Letournel; (4) post-ORIF with anatomical reduction; and (5) need for follow-up for more than 1 year. The exclusion criteria were (1) concomitant intracranial hemorrhage requiring surgery or cerebrovascular accident; (2) advanced hip-joint osteoarthritis (OA) or femoral head osteonecrosis (ON) as indicated on preoperative radiographs; and (3) PWFs with poor reduction quality showing a postoperative fracture gap > 3 mm, assessed using Matta’s criteria. After checking the inclusion and exclusion criteria, a total of 60 patients were enrolled in this study.

### 2.2. Preoperative Assessments and Treating Methods

**Figure 1:**
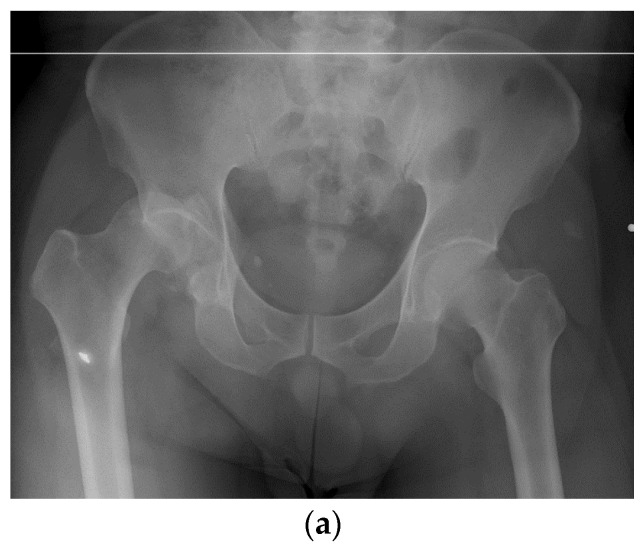

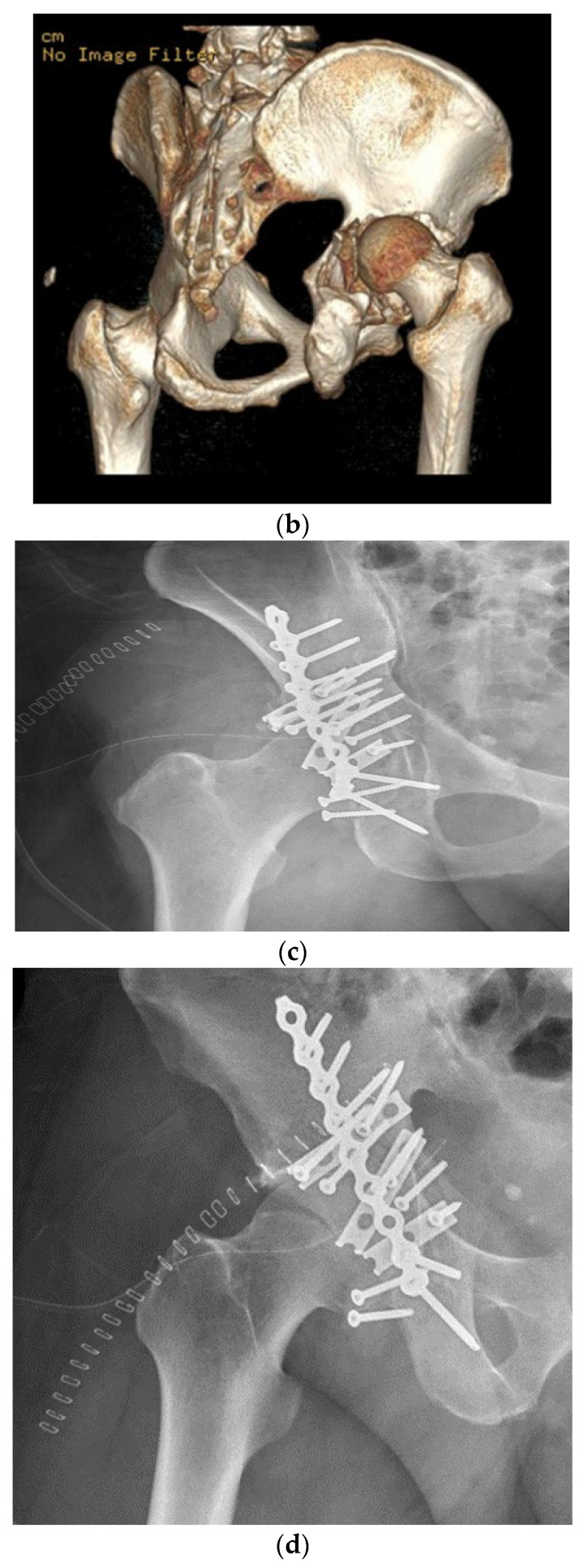
A 55-year-old male patient with injury to the right hip in a traffic accident. Preoperative radiography and CT scan reveal comminuted posterior right acetabular wall fracture associated with dome comminution (**a**,**b**). Open reduction and internal fixation with additional three spring plates have been performed to stabilize the comminuted fragments. Postoperative radiography shows good reduction of the acetabular joint surface (**c**,**d**).

All patients had undergone standard preoperative treatment and examination. Skeletal traction was routinely applied while awaiting surgery to avoid further injury from joint motion. Standard preoperative radiographic examinations included antero-posterior, lateral, and two 45° oblique Judet views of the injured hip. Routine preoperative CT scans were performed to facilitate detailed fracture pattern evaluation, intra-articular fragment detection, and detailed surgical planning. All surgeries were conducted by two experienced senior hip surgeons using the Kocher-Langenbeck approach. The hip joint structure was inspected by gentle traction, and intra-articular bone fragments were removed if present. The marginal impaction of the posterior acetabular wall was elevated and then reduced using the femoral head as a template. Bone voids were firmly impacted with autologous cancellous bone that was harvested from the greater trochanter. Bone fragments were temporally fixed using K-wires. The reduction quality and K-wire position were verified using intra-operative fluoroscopy. After the confirmation of joint congruity, lag screws were used to secure the bone fragments and replace K-wires. The lag screw positions were rechecked and confirmed to be in the extra-articular position. A buttress reconstruction plate or an additional spring plate was used to buttress the PWFs (Figure 1). After surgery, muscular strength exercises, range of motion exercises, and gait training were allowed under the supervision of a physical therapist. Each patient was asked to bear no weight on the operated limb for 3 months. Progressive weight-bearing walking was allowed according to the results of the following images.

### 2.3. Clinical Outcome Evaluations

Clinical records, including patient features and clinical presentations, were retrospectively reviewed. Postoperative follow-up examinations were performed to assess the clinical and radiologic outcomes at 1, 3, 6, and 12 months and yearly thereafter. The clinical outcomes for hip function were assessed using the Harris hip score (HSS) and the modified Merle d’Aubigné clinical hip score (MMAS). The maximum HSS is 100, and results can be interpreted as poor (<70), fair (70–79), good (80–89), or excellent (90–100). The MMAS was graded as poor (3–11), fair (12–14), good (15–17), or excellent (18). In this study, poor clinical results were defined as HSS < 80 and MMAS < 14.

### 2.4. Radiological Outcome Assessments

Preoperative and final follow-up radiographs were cross checked by two surgeons who were not involved in the surgery to evaluate the fracture pattern, reduction quality, and presence of posttraumatic OA or femoral head ON. Postoperative CT scans were not routinely used except in cases of suspected screw malpositions. Fracture comminution was defined as the presence of three or more fragments on CT. Fracture comminution was further divided into two groups according to the presence or absence of acetabular dome comminution. Final follow-up radiographs were used to grade the stage of hip OA according to the Kellgren--Lawrence scale and to classify the stage of femoral head ON using the criteria of Ficat and Arlet. Poor radiographic outcomes included the presence of advanced OA (stage 3/4) and ON (stage 3/4) or the need for conversion to THA.

### 2.5. Statistical Analysis

Data are expressed as mean ± standard deviation and were processed by an expert statistician using SPSS software (version 20.0, SPSS Inc., Chicago, IL, USA). Continuous variables between the groups were compared using Student’s t-test or analysis of variance, as appropriate. Categorical variables were analyzed using the chi-square test or Fisher’s exact test, as appropriate. Spearman’s rho (correlation coefficient) between age and functional scores was calculated using Spearman’s rank correlation. The functional scores in patients of different age groups with comminuted PWFs were compared using a general linear regression model. A post hoc analysis was performed using the Bonferroni test.

## 3. Results

Pre- and postoperative clinical and radiographic results are shown in Table 1. A total of 16 women (26.7%) and 44 men (73.3%) with an average age of 39.1 years were included in this study. Only 18.3% of PWFs comprised a single large fragment without other radiographic factors. The majority of PWFs were multifragmentary (61.7%) and usually associated with hip dislocation (41.7%), acetabular dome comminution (31.7%), marginal impaction (18.3%), and femoral head injury (21.7%). Most patients achieved functional performance after surgery with excellent to good results (83.3% and 80% on the HHS and MMAS, respectively). The final radiographs revealed advanced OA, ON with femoral head collapse, and the requirement of THA in 18.3%, 8.3%, and 10% of patients, respectively.

**Figure 2:**
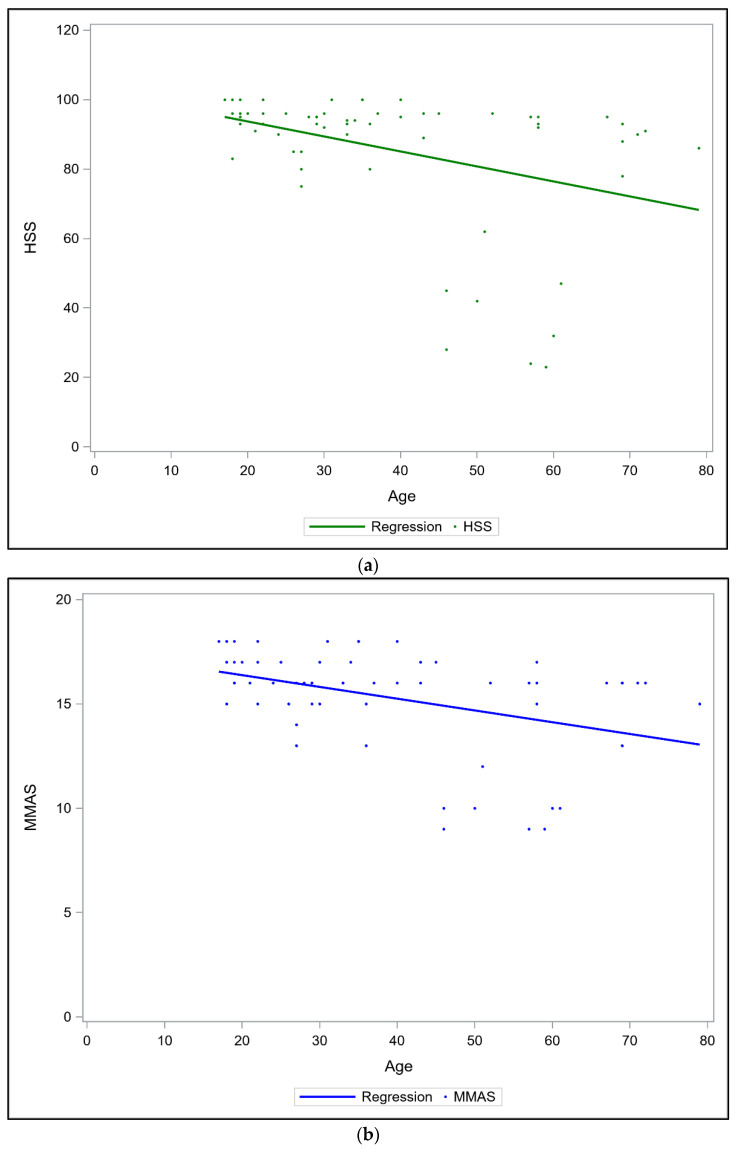
The correlations between patient age and postoperative functional scores. The Spearman rank correlations are −0.41 in (**a**) HHS (*p* < 0.0012) and −0.39 in (**b**) MMAS (*p* = 0.0019), indicating a negative moderate correlation between patient age and functional scores.

Age was found to be significantly negatively correlated with functional results in terms of HHS (−0.41; *p* = 0.0012) and MMAS (−0.39; *p* = 0.0019) (Figure 2). Common pre-operative CT scans revealed that fragment comminution of either the posterior wall or dome region was significantly associated with poor functional scores. Other factors, including hip dislocation, marginal impaction, and femoral head injury, were not significantly related to functional outcomes (Table 2). Patients with fragment comminution showed significantly lower postoperative functional scores than those without comminution (18.2% and 15.4% difference in the mean HHS and MMAS, respectively; both p < 0.0001). Fracture comminution involving the dome was further associated with a lower HHS (reduction of 27.5%) and MMAS (reduction of 20.7%) than fracture comminution without dome area involvement.

Age, fracture comminution, and dome comminution were significantly associated with poor radiographic outcomes (Table 3). The presence of fragment comminution was significantly related to the occurrence of advanced OA. Furthermore, both age and acetabular dome comminution were associated with a higher risk of advanced OA and ON, as well as the need for THA.

To analyze the effects of fragment comminution, we classified patients into three groups according to dome area involvement: group 1 (PWFs without fragment comminution), group 2 (PWFs with fragment comminution but no dome area involvement), and group 3 (PWFs with acetabular dome comminution). The clinical functional scores worsened from groups 1 to 2 to 3. Patients with comminuted PWFs involving the dome (group 3) exhibited significantly lower HHS (*p* < 0.0001) and MMAS (*p* < 0.0001) than those in the other groups (Table 4 and Table 5). In addition to the functional scores, there was a significant difference in the development of advanced OA and the need for conversion to THA among the groups (Table 4 and Table 5). Post hoc comparisons further indicated that acetabular dome comminution was the most critical prognostic factor. Patients with dome comminution exhibited the lowest average functional scores (both HHS and MMAS) and had a higher risk of advanced OA and need for THA than those with fragment comminution without dome involvement.

## 4. Discussion

Anatomical reduction is the most fundamental determinant of good clinical outcomes in PWFs. However, some patients with PWFs still have poor postoperative function even after the anatomical reduction of the fracture. In our study, we comprehensively analyzed the relevance of possible patient factors and fracture pattern factors to both clinical and radiographic outcomes after the anatomical reduction of isolated acetabular PWFs. We found that age, fracture comminution, and dome comminution were significantly related to clinical and radiographic outcomes. Age, a significant risk factor, showed negative correlations with functional scores (HHS and MMAS) and radiographic changes. Furthermore, patients with fracture comminutions involving the weight-bearing zone of the acetabulum presented significantly lower HHS and MMAS and poorer radiographic outcomes (advanced OA, ON, and THA) than those without dome involvement.

Some prognostic factors for outcomes in patients after surgical treatment of PWFs have been reported in the literature; however, most studies discuss outcomes in all types of acetabular fractures [4,11,12]. A few studies exclusively evaluating PWFs have indicated that fractures occurring at a later age with intra-articular comminution or with marginal impaction mainly contributed to adverse effects and emphasized the importance of anatomical reduction [3]. The quality of reduction affects the prognosis of acetabular fractures; however, some fractures are too comminuted to achieve anatomical reduction, resulting in a poor prognosis. Moreover, some patients may experience fracture displacement after undergoing an initial anatomical reduction. Unsatisfactory clinical outcomes of PWFs that were perfectly reduced initially are not uncommon. Factors associated with adverse effects in PWFs that were initially anatomically reduced remain unclear. In this study, we evaluated only PWFs with radiographic anatomical reduction to eliminate the factors of surgical impropriety and aimed to explore the prognostic factors that could affect postoperative clinical and radiographic outcomes. We demonstrated that dome comminution was the most significant factor associated with poor functional scores, advanced OA, and the need for THA.

Extensive fracture comminution makes it difficult to achieve anatomical reduction and is more likely to lead to poor clinical results [13]. We excluded PWFs without radiographic anatomical reduction and focused on the significance of the area where the fracture was comminuted. The acetabular dome supports a tremendous weight-bearing load; hence, a fracture involving the weight-bearing dome is considered a risk factor for poor clinical outcomes. Ovre et al. retrospectively reviewed 450 acetabular fractures to analyze the relationship between clinical outcome and the roof arc angle (angle between a vertical line drawn from the center of the acetabulum toward the acetabular dome and a second line drawn from the center of the acetabulum to the fracture) converted to a roof arc score. Their results indicated the significance of fracture lines in the acetabular dome to clinical outcomes. Our results showed a significant association between dome comminution and advanced OA. The acetabular dome is subjected to great force during movement; therefore, a single buttress plate fixation may not be sufficient to stabilize the comminuted bone and prevent its displacement. The residual steps and diastasis causing joint incongruence on the weight-bearing dome seemed to be correlated with poor clinical outcomes and an increase in the risk of OA, femoral head ON, and conversion to THA. THA for the treatment of failed acetabular fractures is a challenging procedure that usually results in inferior functional outcomes and higher complication rates than primary THA. Recently, experts have shown interest in performing THA for early rehabilitation in patients with femoral head impaction, preexisting OA or ON, or osteoporotic bone with comminution in the acetabular dome [14]. However, the risk of heterotopic ossification (HO) in THA patients may hamper rapid rehabilitation [15]. Moreover, the incidence of HO was reported to be higher in patients undergoing acute THA for acetabular fractures and contributed to adverse functional outcomes [16].

Age has been demonstrated to be a negative prognostic factor for acetabular fractures. The poor bone quality and higher rate of fragment comminution and marginal impaction in patients with advanced age preclude a secure fixation and lead to early implant failure and a higher rate of conversion to THA [17,18]. Hence, some authors have suggested immediate THA for geriatric comminuted acetabular fractures for early mobilization [19]. Extensive research has been conducted on all types of acetabular fractures in elderly patients; however, studies focusing on PWFs in older patients are scarce. Ferguson et al. reviewed 31 PWFs from 235 geriatric acetabular fractures and found that 64% of fractures were comminuted and 52% were combined with marginal impaction; however, they did not report their associations with clinical outcomes [18]. The results from another review article including 15 studies on acetabular fractures in patients aged over 55 years indicated an average conversion rate to THA of 23.1% (0% to 45.5%) at a mean follow-up of 47.3 months [20]. As reported in previous studies, we noted a higher rate of comminution (66.7%, 6/9) in patients older than 60 years; additionally, age correlated negatively with both clinical and radiographic outcomes even after anatomical reduction.

Marginal impaction injuries represent chondral depressions in PWFs in which an osteochondral fragment is rotated and impacted into the underlying subchondral bone [8]. Studies have reported that patients with PWFs and marginal impaction showed poor clinical outcomes because of the failure to identify the improperly reduced impacted fragments [11]. With the advances in surgical techniques and the awareness of fracture patterns from preoperative CT scans, the clinical outcomes of PWFs with marginal impaction have improved [10,21]. Preoperative CT scan provides more precise information about the status of margin impaction in an acetabular fracture and helps surgeons to ensure accurate fracture reduction, thereby preventing possible complications. A two-level reconstruction of marginal impaction, including the reduction of the osteochondral fragment in an anatomical position using the femoral head as a template and impacting the void cavity with an autologous bone graft or freeze-dried allograft, can improve postoperative clinical outcomes [11]. In this study, routine preoperative CT scans were performed to precisely inspect defects in marginal impaction, and a two-level reconstruction technique was used to ensure accurate reduction in patients with PWFs. Although there were no significant differences in functional outcomes or THA rates, PWFs with marginal impaction still exhibited a higher risk of radiographic OA.

In this study, we evaluated PWFs with radiographic anatomical reduction to eliminate factors of surgical impropriety. We demonstrated that dome comminution was the most significant factor associated with poor outcomes. As a result of the tremendous load on the acetabular dome and the inaccurate evaluation of bone union from radiography, these patients may require longer periods of activity restriction and nonweight-bearing exercises. We also inferred that an additional spring plate over the dome region may be beneficial for securing comminuted fragments. Studies on the use of an additional spring plate over the dome area are ongoing.

Our study has certain limitations. First, the study was retrospective and included only a limited number of patients, particularly elderly patients; therefore, the statistical conclusions of the relational comparisons should be interpreted cautiously. Second, the follow-up period was relatively short and did not reveal the potential long-term complications associated with OA progression. With the increasing incidence of acetabular fractures in geriatric patients, conversions to THA are increasing. Understanding the risk factors of failed treatment of acetabular fractures in the elderly population is essential for orthopedic surgeons to reduce complications after ORIF. Additional studies with larger samples of elderly patients and longer observation periods are warranted for clarifying the potential risk factors for postoperative complications and poor clinical outcomes. Third, postoperative reduction was evaluated with X-ray images, which may result in under-diagnosis of inadequate reduction. Although postoperative CT scans provide the precise evaluation of fracture reduction, it was reported that benefits were limited to patients after the fixation of acetabular fractures [22]. There is still no consensus on the use of routine pelvic CT after surgery. Fourth, we evaluated only the impacts of fracture-related factors on prognosis, and we did not consider differences in individuals’ soft tissues. Soft tissue composition before injury and degree of soft tissue injury may also have a significant impact on functional outcomes. A close relationship has been reported between hip OA and changes in the collagen content in the fascia lata. The increase in collagen type I along with a decrease in collagen type III and hyaluronan levels results in facial stiffening, which may increase the risk of OA [23].

## 5. Conclusions

Age, fracture comminution, and dome comminution were the prognostic indicators of advanced OA and poor functional scores even after anatomical reduction. We emphasized the relevance of acetabular dome comminution as an important contributing factor to poor clinical and radiographic outcomes. Surgeons should be aware of the high complication rates with dome comminution and warn patients of the high incidence of poor outcomes. Although we noted a high complication rate of such fractures, the best treatment strategy for them is currently unknown. Further studies are needed to validate whether using an additional spring plate over the acetabular dome or performing THA in the acute phase can provide a better prognosis than traditional buttress fixation.

## Figures and Tables

**Table 1:** The patients’ preoperative and postoperative clinical and radiographic data.

	Total Patients
N	60
Age, years	
Mean (SD)	39.1(17.3)
Sex, *n* (%)	
Female	16 (26.7)
Male	44 (73.3)
Preoperative CT evaluation, *n* (%)	
Fracture comminution	37 (61.7)
Dome comminution	19 (31.7)
Dislocation	25 (41.7)
Marginal impaction	11 (18.3)
Femoral head injury	13 (21.7)
Clinical function outcomes	
HSS	
Mean (SD)	85.5 (20.1)
MMAS	
Mean (SD)	15.3 (2.5)
Radiographic outcomes, *n* (%)	
OA	
Early stage (stage 0–2)	49 (81.7)
Advanced stage (stage 3–4)	11 (18.3)
ON	
Early stage (stage 0–2)	55 (91.7)
Advanced stage (stage 3–4)	5 (8.3)
Convert to THA	
No	54 (90.0)
Yes	6 (10.0)

Values are percentage (%), standard deviation (SD), interquartile range (IQR). HSS, Harris hip score; MMAS, modified Merled’Aubigné-Postel score; OA, osteoarthritis; ON: osteonecrosis; THA: total hip arthroplasty.

**Table 2:** The prognostic factors related to clinical outcomes (HHS and MMAS).

	HSS	*p*	MMAS	*p*
Age, Spearman’s rho	−0.41	0.0012 *	−0.39	0.0019 *
Sex, Median (IQR)				
Female	92.5 (89.0–96.0)		16.0 (15.0–17.0)	
Male	93.5 (85.0–96.0)	0.9665	16.0 (15.0–17.0)	0.7110
Preoperative radiographic factors, Median (IQR)				
Fracture comminution				
No	96.0 (95.0–100.0)		17.0 (16.0–18.0)	
Yes	90.0 (78.0–93.0)	<0.0001 *	15.0 (13.0–16.0)	<0.0001 *
Dome comminution				
No	95.0 (93.0–96.0)		16.0 (16.0–17.0)	
Yes	80.0 (42.0–91.0)	<0.0001 *	13.0 (10.0–16.0)	<0.0001 *
Dislocation				
No	95.0 (88.0–96.0)		16.0 (16.0–17.0)	
Yes	92.0 (80.0–94.0)	0.0765	16.0 (14.0–16.0)	0.0717
Marginal impaction				
No	94.0 (86.0–96.0)		16.0 (15.0–17.0)	
Yes	91.0 (83.0–93.0)	0.1267	15.0 (15.0–16.0)	0.1805
Femoral head injury				
No	94.0 (88.0–96.0)		16.0 (15.0–17.0)	
Yes	92.0 (85.0–95.0)	0.3166	16.0 (15.0–16.0)	0.2553

HSS, Harris hip score; MMAS, modified Merled’Aubigné-Postel score. * *p* < 0.05.

**Table 3:** The prognostic factors related to radiographic outcomes (OA, ON and THA).

	OA			ON			THA		
	Early Stage	Advanced Stage	*p*	Early Stage	Advanced Stage	*p*	No	Yes	*p*
*n*	49	11		55	5		54	6	
Age,									
Mean (SD)	36.4 (17.3)	51.1 (12.1)	0.0100 *	37.8 (17.4)	53.8 (7.3)	0.0468 *	37.6 (17.5)	53 (6.4)	0.0374 *
Median (IQR)	31.0 (22.0–43.0)	51.0 (46.0–60.0)	0.0057 *	33.0 (24.0–51.0)	57.0 (46.0–59.0)	0.0302 *	33.0 (24.0–51.0)	53.5 (46.0–59.0)	0.0226 *
Sex female, *n* (%)	15 (30.6)	1 (9.1)	0.2586	16 (29.1)	0 (0.0)	0.3113	15 (27.8)	1 (16.7)	1.0000
Fracture comminution	26 (53.1)	11 (100.0)	0.0042 *	32 (58.2)	5 (100.0)	0.1460	31 (57.4)	6 (100.0)	0.0733
Dome comminution	9 (18.4)	10(90.9)	<0.0001 *	15 (27.3)	4 (80.0)	0.0312 *	13 (24.1)	6 (100.0)	0.0005 *
Dislocation	18 (36.7)	7 (63.6)	0.1744	22 (40.0)	3 (60.0)	0.6405	21 (38.9)	4 (66.7)	0.2234
Marginal impaction	9 (18.4)	2 (18.2)	1.0000	10 (18.2)	1 (20.0)	1.0000	10 (18.5)	1 (16.7)	1.0000
Femoral head injury	10 (20.4)	3 (27.3)	0.6899	12 (21.8)	1 (20.0)	1.0000	12 (22.2)	1 (16.7)	1.0000

OA, osteoarthritis; ON: osteonecrosis; THA: total hip arthroplasty. * *p* < 0.05.

**Table 4:** The effects of different status of comminution on clinical and radiographic outcomes in patients with PWF.

	Group 1	Group 2	Group 3	
	No Fragment Comminution	Fragment Comminution but Not Dome Comminution	Acetabular Dome Comminution	*p*
Total patients, *n*	23	18	19	
HSS, LS-mean (SE) **	93.87 (3.11)	92.64 (3.50)	68.45 (3.33)	<0.0001 *
MMAS, LS-mean (SE) **	16.62 (0.35)	15.98 (0.39)	13.06 (0.38)	<0.0001 *
OA, *n* (%) †,‡				
Early stage	23 (100.0)	17 (94.4)	9 (47.4)	<0.0001 *
Advanced stage	0 (0.0)	1 (5.6)	10 (52.6)	
ON, *n* (%) †,‡				
Early stage	23(100.0)	17 (94.4)	15 (78.9)	0.0333 *
Advanced stage	0 (0.0)	1 (5.6)	4 (21.1)	
THA, *n* (%) †,‡				
No	23(100.0)	18 (100.0)	13 (68.4)	0.0009 *
Yes	0 (0.0)	0 (0.0)	6 (31.6)	

SE: standard error; LS-mean: Least-squares means. Group 1: no fragment comminution; Group 2: fragment comminution but not dome comminution; Group 3: acetabular dome comminution. HSS, Harris hip score; MMAS, modified Merled’Aubigné-Postel score; OA, osteoarthritis; ON: osteonecrosis; THA: total hip arthroplasty; * *p* < 0.05, ** Data set was calculated after the adjustment of age using a generalized linear regression model. † *p* values were calculated with the use of a Fisher’s exact test to compare fracture comminution with clinical and radiographic outcomes. ‡ Adjusted odds ratio (OR) with 95% confidence interval (CI) was estimated after adjustment for age using multinomial logistic regression.

**Table 5:** Post hoc comparisons for the effects of different status of comminution on clinical and radiographic outcomes in patients with PWF.

Post hoc Analysis	Group 1 vs. Group 2		Group 1 vs. Group 3		Group 2 vs. Group 3	
	Difference LS-Mean (SE) /OR (95% CI)	*p*	Difference LS-Mean (SE) /OR (95% CI)	*p*	Difference LS-Mean (SE) /OR (95% CI)	*p*
Total patients, *n*						
HSS, LS-mean (SE) **	−1.24 (4.80)	1.0000	−25.43 (4.58)	<0.0001*	−24.19 (4.81)	<0.0001 *
MMAS, LS-mean (SE) **	−0.64 (0.54)	0.4769	−3.56 (0.52)	<0.0001*	−2.92 (0.54)	<0.0001 *
OA, *n* (%) †,‡						
Early stage	1.00		1.00		1.00	
Advanced stage	4.03 (0.15–104.93)	0.4390	51.95 (2.76–978.12)	<0.0001*	58.96 (3.90–891.8)	0.0033 *
ON, *n* (%) †,‡						
Early stage	1.00		1.00		1.00	
Advanced stage	4.03 (0.15–104.93)	0.4390	13.65 (0.69–271.70)	0.0346*	6.43 (0.60–69.33)	0.1250
THA, *n* (%) †,‡						
No	1.00		1.00		1.00	
Yes	-	-	-	-	22.63 (1.18–433.78)	0.0052 *

SE: standard error; LS-mean: Least-squares means. Group 1: no fragment comminution; Group 2: fragment comminution but not dome comminution; Group 3: acetabular dome comminution. HSS, Harris hip score; MMAS, modified Merled’Aubigné-Postel score; OA, osteoarthritis; ON: osteonecrosis; THA: total hip arthroplasty; * *p* < 0.05, ** Data set was calculated after the adjustment of age using a generalized linear regression model. † *p* values were calculated with the use of a Fisher’s exact test to compare fracture comminution with clinical and radiographic outcomes. ‡ Adjusted odds ratio (OR) with 95% confidence interval (CI) was estimated after adjustment for age using multinomial logistic regression.

## Data Availability

The datasets generated and analyzed during the current study are available from the corresponding author on reasonable request.
